# Prevalence and course of pregnancy symptoms using self-reported pregnancy app symptom tracker data

**DOI:** 10.1038/s41746-023-00935-3

**Published:** 2023-10-11

**Authors:** Michael Nissen, Nuria Barrios Campo, Madeleine Flaucher, Katharina M. Jaeger, Adriana Titzmann, Dominik Blunck, Peter A. Fasching, Victoria Engelhardt, Bjoern M. Eskofier, Heike Leutheuser

**Affiliations:** 1 Machine Learning and Data Analytics (MaD) Lab, Department Artificial Intelligence in Biomedical Engineering, Friedrich-Alexander-Universität Erlangen-Nürnberg, Carl-Thiersch-Straße 2b, 91052 Erlangen, Bavaria Germany; 2 Department of Gynecology and Obstetrics, Erlangen University Hospital, Friedrich-Alexander-Universität Erlangen-Nürnberg, Universitätsstraße 21/23, 91054 Erlangen, Bavaria Germany; 3 Department of Health Management, Institute of Management, Friedrich-Alexander-Universität Erlangen-Nürnberg, Lange Gasse 20, 90403 Nürnberg, Bavaria Germany; 4 Keleya Digital-Health Solutions GmbH, Max-Beer-Straße 25, 10119 Berlin, Germany; 5 Translational Digital Health Group, Institute of AI for Health, Helmholtz Zentrum München - German Research Center for Environmental Health, Ingolstädter Landstraße 1, 85764 Neuherberg, Bavaria Germany

**Keywords:** Reproductive signs and symptoms, Pathogenesis, Medical research, Computational biology and bioinformatics

## Abstract

During pregnancy, almost all women experience pregnancy-related symptoms. The relationship between symptoms and their association with pregnancy outcomes is not well understood. Many pregnancy apps allow pregnant women to track their symptoms. To date, the resulting data are primarily used from a commercial rather than a scientific perspective. In this work, we aim to examine symptom occurrence, course, and their correlation throughout pregnancy. Self-reported app data of a pregnancy symptom tracker is used. In this context, we present methods to handle noisy real-world app data from commercial applications to understand the trajectory of user and patient-reported data. We report real-world evidence from patient-reported outcomes that exceeds previous works: 1,549,186 tracked symptoms from 183,732 users of a smartphone pregnancy app symptom tracker are analyzed. The majority of users track symptoms on a single day. These data are generalizable to those users who use the tracker for at least 5 months. Week-by-week symptom report data are presented for each symptom. There are few or conflicting reports in the literature on the course of diarrhea, fatigue, headache, heartburn, and sleep problems. A peak in fatigue in the first trimester, a peak in headache reports around gestation week 15, and a steady increase in the reports of sleeping difficulty throughout pregnancy are found. Our work highlights the potential of secondary use of industry data. It reveals and clarifies several previously unknown or disputed symptom trajectories and relationships. Collaboration between academia and industry can help generate new scientific knowledge.

## Introduction

Throughout pregnancy, almost all women experience pregnancy-related symptoms^1,2^. Prior work identified up to 41 different symptoms^3,4^. Reported incidences in literature highly differ. Common symptoms include fatigue (up to 98%)^3–6^, nausea (up to 88%)^1–4,6–9^, poor sleep (up to 74%)^3,4,6,8^, back pain (up to 60%)^1,3,4,7^, and vomiting (up to 57%)^1,2,4,6–9^.

Pregnancy symptoms can cause discomfort for women, decrease the quality of life^10–12^, and lead to higher socioeconomic costs due to prolonged sick leave^13,14^. Furthermore, several pregnancy symptoms are associated with adverse outcomes for mothers and children: The risk of preterm delivery, lower birth weight, consequences for infant development, neonatal wellbeing, and obstetric complications are significantly linked to depression, anxiety, and maternal stress^15–18^. Furthermore, significant associations exist between individual symptoms, such as depression, anxiety, increased nausea, and vomiting^13,19^. Vaginal bleeding between 6 and 8 weeks’ gestation can result in an increased risk of pregnancy loss^20^. Severe nausea and vomiting in pregnancy are associated with higher hospitalizations and negative outcomes^21,22^. Insomnia and sleep disturbances are linked to increased rates of cesarean delivery, preterm birth, and postpartum depression^23–28^.

The overall relationship between pregnancy symptoms as well as their relation to clinical outcomes is not well understood^19,23,29,30^. Previous work explicitly calls for big data sources with detailed collected information on multiple signs and symptoms, data from pregnancy mobile health apps, as well as more accurate reporting^29^.

The integration of digital health, particularly mobile health apps, into clinical trials and research has several benefits: It can facilitate recruitment, reach broader populations, and provide real-world data^31^. Previous studies on pregnancy symptoms have been mostly collected retrospectively using questionnaires during hospital visits^29^. Retrospective symptom assessment is often biased compared to momentary assessment^32^.

The number of pregnancy apps exceeds the market for all other medical topics^33^. In previous pregnancy app reviews, all of the assessed applications were of commercial nature^34^, and the minority of apps mentions an involvement of medical experts^35^. Many applications feature a large user base and offer tools such as symptom trackers. These may still be helpful to generate new scientific insights, such as in the context of pregnancy symptoms and outcomes. Meanwhile, real-world data is often messy, heterogeneous, and requires new analysis techniques^36^.

This work analyzes a data set originating from the symptom tracker of the Keleya pregnancy app (Keleya, Keleya Digital-Health Solutions GmbH, Berlin, Germany). This app is designed to meet the needs of pregnant women in German-speaking countries. It aims to support women on their pregnancy and motherhood journey. Keleya promotes a collaboration with medical experts and cooperates with several health insurances. Features included are information about the current week of pregnancy, recipes, workouts, meditation, breastfeeding advice, and a symptom tracker.

We report the results of a retrospective analysis of 1,549,186 symptom reports, from 183,732 users and subsets thereof. To our knowledge, this is the most comprehensive work on pregnancy symptoms to date and exceeds previous works.

This work has two main goals: First, to present methods to handle noisy, real-world app data from commercial health applications to understand the prevalence and trajectory of user and patient-reported data. Second, to examine symptom report percentages, their changes over time, and their correlation at a detailed level. We then discuss collaboration between industry and academia as a tool to generate real-world evidence, as well as the resulting implications.

## Results

### User definition and subpopulation selection

In traditional clinical studies, participants are often actively approached and recruited by physicians. In contrast, pregnancy app users often choose to use pregnancy apps at their own choice, often without intervention or impulse by their care practitioners. Similarly, there is no structured follow-up. Use, and consecutively generation of research data, is entirely dependent on users’ intrinsic motivation to use apps and can vary widely.

Therefore, defining an appropriate subpopulation for consecutive analysis is a major challenge. In the analyzed data set, many users only used the symptom tracker feature once, and it is unclear whether the reported data are accurate in this case.

The application of a fixed threshold for the definition of “active users” was not useful. We found that the selection of subpopulations should be guided by three pillars: The analysis goals, time frame of interest, and data distribution/properties. In this work, the analysis goals are an estimation of symptom prevalence, symptom changes over time, and symptom correlation. The time frame of interest is the whole pregnancy duration and the postpartum period. The data characteristics are described in the next subchapter. The choice of the most appropriate user definition for analysis can only be made after an assessment of the characteristics of the subpopulation. For this work, we ideated 14 different definitions for subgroups criteria for data selection. These are shown in Table 1.

**Table 1:** Symptom tracker data set characteristics (unique users, total tracked symptoms, mean and standard deviation of reported symptoms per user, mean and standard definition of usage time in days) for different data selection (inclusion/exclusion) criteria.

Data Set Description	Users	Symptoms	Symptoms/User	Usage time
A: All data	183,732	1,549,186	8.44 ± 16.68	17.18 ± 68.26
B: Only symptoms from first tracker use	183,732	606,705	3.30 ± 2.61	0.00 ± 0.00
C: Only symptoms from first day of tracker use	183,732	960,653	5.23 ± 3.89	0.00 ± 0.03
D: Only users that tracked minimum 2 symptoms	168,225	1,533,679	9.12 ± 17.27	18.76 ± 71.13
E: Only users tracking on a single day	145,336	763,469	5.25 ± 3.83	0.00 ± 0.02
F: Excl. symptoms from first tracker use	99,838	942,481	9.45 ± 21.61	23.64 ± 78.75
G: Only users tracking symptoms on min. 2 diff. days	38,396	785,717	20.48 ± 33.06	82.18 ± 130.21
H: Excl. symptoms from first day of tracker use	38,396	588,533	15.34 ± 32.32	48.06 ± 110.76
I: Excl. symptoms tracked in first 3 days after registration	38,375	564,613	14.72 ± 31.90	45.58 ± 109.57
J: Excl. symptoms tracked in first week after registration	35,119	525,522	14.97 ± 32.42	46.89 ± 111.96
K: Only users tracking symptoms in min. 2 diff. trimesters	18,596	527,168	28.37 ± 44.09	138.27 ± 150.45
L: Only users tracking symptoms in min. 4 diff. weeks	10,182	457,284	44.95 ± 55.99	157.53 ± 171.07
M: Only users tracking min. 1 symptom in all trimesters	3735	210,729	56.48 ± 74.66	241.79 ± 198.81
N: Only users tracking symptoms in min. 5 diff. months	2919	226,351	77.62 ± 85.14	246.16 ± 212.99

The respective data selection criteria and consecutive data sets are denoted by the corresponding letters in the remainder of this work. Symptoms/user is reported as mean ± standard deviation. Usage time is defined as difference between first and last symptom tracker use. Usage time is reported per user in days, mean ± standard deviation.

### Usage and tracked symptoms

The number of unique users, total number of reported symptoms, the mean and standard deviation of reported symptoms per user, and the mean and standard definition of usage time in days are also reported in Table 1.

After preprocessing, the data set contained symptom reports from 183,732 users with a total of 1,549,186 tracked symptoms, tracked from May 31, 2018 to December 19, 2022. The mean duration between the first and last symptom report (usage time) was 17.18 days. When only users that tracked more than one symptom are used as criteria, the data set characteristics are similar.

The use of stricter criteria for selecting the data set results in a lower number of included users and total tracked symptoms, while the mean value of reported symptoms per user and usage time increases.

**Fig. 1:**
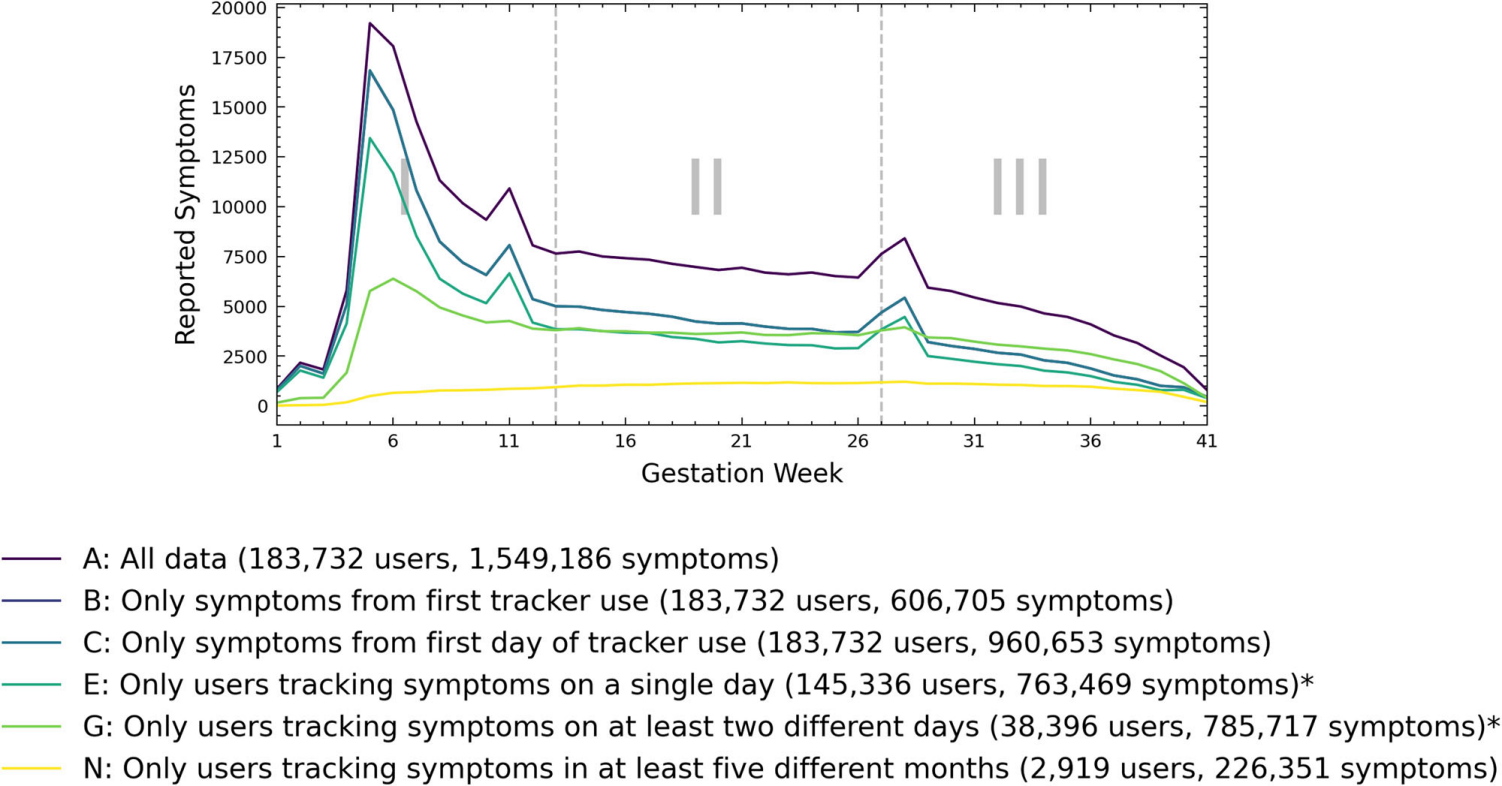
Total number of symptoms recorded each week, for selected data set selection criteria. Some data set selection criteria have been omitted for clarity. See Table 1 for details on the respective data selection criteria. The highest number of symptoms is recorded around gestation weeks 4–5.

Figure 1 shows the weekly total number of reported symptoms based on different data set definitions. Reported symptoms increase sharply around 3–4 weeks of gestation. Peaks in weekly reported symptoms are observed around gestation weeks 4–5, 11, and 28 when weaker data selection criteria are considered.

### User demographics

Due to the anonymized nature of the data set, only limited demographic information is available. The app’s developer, Keleya, applies the principle of data minimization, which further reduces the available information. 83% of the users use the German version of the application, 17% of users use the English version. The users are equally divided (50%) between iOS and Android. Based on a previous user survey (*n* = 157) by Keleya, 70% (*n* = 110) of their users are in their first pregnancy. Most users are 31–39 years old (64.3%, *n* = 101), 23.6% are 26–30 years old (*n* = 37), 7% are 40 or older (*n* = 11), and 5.1 % are 18–25 years old (*n* = 8).

### Symptom occurrence and frequency

Individual symptom occurrences for each data selection criteria are listed in Table 2. Symptom percentages differ greatly for different data selection criteria. Stricter data selection criteria result in higher percentages overall. The data selection criterion “all users” (selection criterion A) shows fatigue (48.7%), back pain (44.2%), constipation (32.2%), sleeping difficulty (30.1%), and breathlessness (29.0%) as most prevalent symptoms. This order is also largely consistent across other data selection criteria, although breathlessness (81.0%) is more common than sleeping difficulty (79.4%) among “users that tracked symptoms in at least 5 different months” (data selection criterion N). Detailed statistics such as total symptom counts, their occurrence, and frequency for this criterion (selection criterion N) are included in the Supplementary Table 1.

**Table 2:** Symptom occurrence by data selection criteria, across the whole pregnancy.

Symptom	A	B	C	D	E	F	G	J	K	L	M	N
Back Pain	44.2	23.4	38.6	48.2	39.1	44.5	63.3	53.3	73.3	82.8	86.4	92.6
Bladder Weakness	13.0	5.7	10.2	14.1	10.3	14.7	22.9	20.7	30.6	38.9	47.1	53.1
Breathlessness	29.0	13.3	24.1	31.6	23.8	32.2	48.5	40.0	58.0	68.9	74.8	81.0
Constipation	32.2	18.6	27.8	35.2	28.0	29.4	48.2	36.0	55.8	65.6	69.8	76.2
Diarrhea	12.8	6.2	9.8	13.9	9.9	13.1	23.5	17.0	27.2	37.5	41.2	49.0
Fatigue	48.7	36.1	43.0	51.5	42.5	33.0	72.0	52.7	75.8	86.0	90.0	92.9
Flatulence	22.6	9.5	19.4	24.6	19.2	26.6	35.1	22.8	37.5	45.0	48.5	49.7
Foot Pain	6.6	2.0	5.1	7.3	5.2	8.9	11.9	9.5	15.4	19.9	24.3	26.4
Headache	25.1	11.9	20.6	27.4	20.8	26.6	41.5	30.3	48.4	61.1	64.2	72.7
Heartburn	23.4	12.4	19.4	25.5	19.7	23.1	37.3	31.4	46.5	55.3	62.8	69.0
Incontinence	16.2	5.8	13.5	17.6	13.9	20.1	24.5	18.0	29.3	36.1	39.7	45.6
Mood: Happy	26.9	17.1	22.3	27.6	22.3	21.9	44.4	31.8	50.0	63.2	65.4	74.0
Mood: Normal	45.2	34.3	39.2	44.8	40.4	27.1	63.3	45.2	69.4	81.4	81.3	89.0
Mood: Scared	13.3	7.2	10.6	14.4	10.6	12.5	23.4	15.7	26.3	36.1	38.6	46.1
Mood: Stressed	15.2	8.1	11.9	16.5	12.1	14.4	26.7	19.2	32.1	42.9	47.1	57.3
Mood: Swings	25.2	15.7	20.7	27.1	21.1	20.4	40.5	29.4	46.4	58.7	61.3	70.3
Nausea	26.1	13.2	21.9	28.4	21.8	26.9	42.1	29.1	45.5	55.2	63.6	65.9
Neck Pain	25.6	12.5	21.5	27.9	21.4	27.2	41.5	31.1	49.0	60.6	64.7	72.9
Nutrition Deficiencies	44.9	28.6	40.5	48.9	41.7	41.1	57.0	43.8	64.9	71.8	76.4	82.0
Pelvic Pain	20.3	7.9	16.1	22.1	16.1	24.6	35.9	29.3	43.9	54.9	61.2	69.0
Sleeping Difficulty	30.1	13.4	25.1	32.9	25.1	34.0	49.1	39.8	56.5	68.5	72.0	79.4

A–N represent different data selection criteria, see Table 1. A represents the data selection criterion with the highest user count, but lowest mean reported symptoms per user. N is the data selection criteria with the lowest user count, but most active users. Symptom occurrences for data selection criterion N (in bold) are most meaningful and should be used for comparison with other works. Occurrences show high differences based on the data selection criteria applied. Flatulence and foot pain symptoms were added during the investigated period. Occurrences for flatulence and foot pain are unreliable and only indicate a lower bound.

### Symptom changes over time

**Fig. 2:**
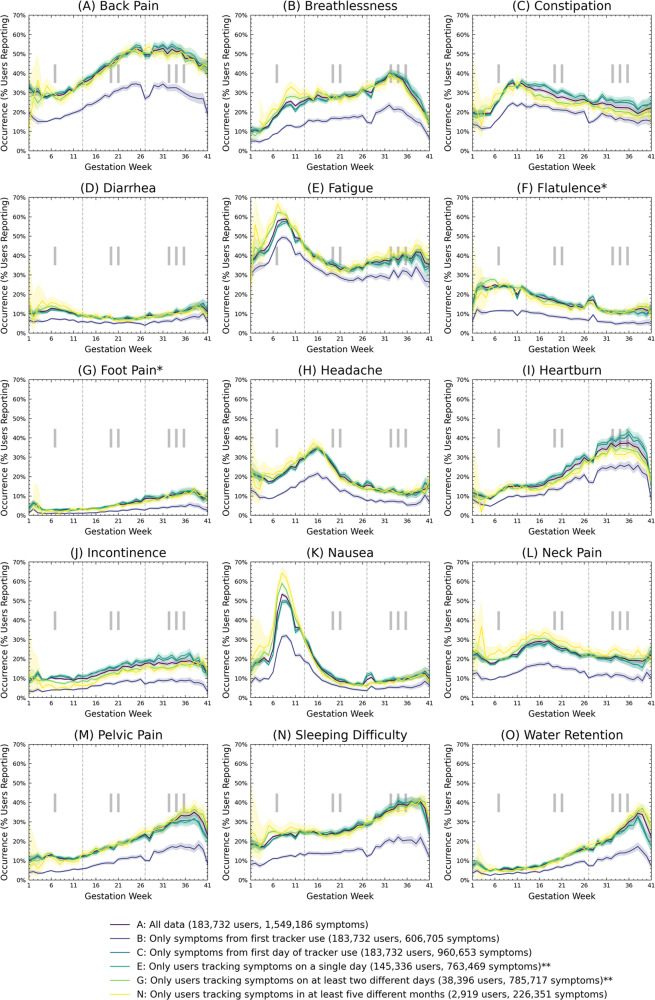
Symptoms progress by gestation week for 15 different pregnancy symptoms. A-O) Week-by-week symptom progress. Each symptom shows clear longitudinal characteristics. Colors depict different data selection criteria, of which some have been omitted for clarity (see Table 1). The offset areas behind each curve show the 95% confidence intervals based on the Agresti-Coull interval (*p* = 0.05). Asterisks indicate symptoms that were added during the analysis period and for which symptom progression is reliable, but occurrence is not. Two asterisks indicate the data selection criteria used to analyze difference significance and effect size.

Figure 2 shows the week-by-week occurrence for each symptom over the duration of pregnancy. Each symptom shows a unique time pattern.

Constipation, fatigue, headache, nausea, and neck pain show clear and distinct peaks around gestation week 7–16. Constipation and neck pain decrease steadily thereafter, while the occurrence of fatigue, headache and nausea increases slightly again toward the end of pregnancy, although much less drastically than before. Back pain has a flat maximum toward the end of pregnancy (around gestation week 24–37). Flatulence is most common at the beginning of pregnancy and decreases steadily toward the end, with a slight spike around gestation weeks 26–28. Diarrhea tends to occur at the beginning and end of pregnancy and has a clear minimum around gestation week 20. Breathlessness, foot pain, heartburn, incontinence, sleeping difficulty, pelvic pain, and water retention increase steadily toward the end of pregnancy, reaching a peak around gestation week 37 and 38. Back pain, breathlessness, constipation, heartburn, pelvic pain show a small local minimum in weekly occurrence around gestation week 27 and 28. Flatulence, headache, and nausea show a small local maximum during these weeks.

The weekly occurrences differ depending on the chosen data selection criterion. The occurrences of “only data from the first tracker usage” (selection criterion B) are always lower than those of the other criteria. The occurrences derived from the other data selection criteria are similar in many cases.

The offset areas behind each curve in Fig. 2 show the 95% confidence intervals based on the Agresti-Coull interval. Furthermore, a *χ*^2^ test was used to test for significant differences between weekly occurrences for all 15 symptoms and 41 weeks. Examined data sets were those of users that used the tracker on one day only (selection criterion E) and those that used the tracker for at least 2 days (selection criterion G). These two data selection criteria are mutually exclusive. No significant difference between the groups was found in 337 (55%) of symptom-week-pairs. An effect size analysis was conducted for all symptom-week-pairs, using the *ϕ* coefficient, odds ratios, and risk ratios. The mean symptom-week effect sizes between the two groups were 0.03 ± 0.02, 1.07 ± 0.2, and 1.05 ± 0.16 (mean ± standard deviation), respectively.

### Symptom correlation

A correlation analysis was conducted using the Pearson correlation coefficient (PCC). The data set derived from the most active users (selection criterion N) were used. Correlations between each symptom for four time periods (each trimester and the Puerperium) were analyzed. The 10 largest absolute correlation coefficients are shown in Table 3. No negative values are included, as they were not among the largest absolute values. Spearman correlation coefficients (SCC) are reported in Supplementary Table 2. The full correlation matrices depicted as heat maps are available in Supplementary Fig. 1 (PCC) and Supplementary Fig. 2 (SCC).

**Table 3:** Ten largest Pearson correlation coefficients (PCC).

Time Frame: Symptom	Time Frame: Symptom	PCC
First: Fatigue	First: Nausea	0.54
Postpartum: Back Pain	Postpartum: Neck Pain	0.53
Postpartum: Constipation	Postpartum: Nutrition Deficiencies	0.53
Postpartum: Bladder Weakness	Postpartum: Mood: Stressed	0.50
Postpartum: Incontinence	Postpartum: Neck Pain	0.46
Postpartum: Bladder Weakness	Postpartum: Breathlessnes	0.45
Postpartum: Bladder Weakness	Postpartum: Sleeping Difficulty	0.43
Postpartum: Fatigue	Postpartum: Pelvic Pain	0.42
Second: Mood: Happy	Third: Mood: Happy	0.41
Postpartum: Diarrhea	Postpartum: Flatulence	0.41

Each symptom in each time period (trimesters, Puerperium) was correlated, using data from the most active users (selection criterion N). No negative values are included, as they were not among the largest absolute values.

## Discussion

We reported findings on the use of a pregnancy app symptom tracker, the symptom occurrences, symptom progress, as well as methods to analyze pregnancy symptom tracker reports. This section is structured as follows: First, the progress of symptoms is discussed in relation to previous work. Then, we discuss symptom tracker engagement, activity, and use. We discuss the methodology and its suitability for this work. We suggest additional reasons for the wide variation in reporting symptom prevalence in the literature. The validity of the symptom tracker used and additional limitations are discussed. Finally, we provide general implications and recommendations for industry and academia.

### Symptom tracker reports are unevenly spread across pregnancy

The total count of reported symptoms for each week (Fig. 1) for the full data set (i.e., without data selection criteria applied) shows three distinct spikes around gestational weeks 6, 10, and 28. Thus, symptoms are not reported uniformly, and the chosen methodology must adjust for this. The first spike coincides with the mean gestational age of pregnancy awareness (5.5 weeks) of US women^37^. This awareness of pregnancy is likely the trigger for downloading and trying pregnancy apps. The spike in week 10 could be related to pregnancy confirmation by a medical provider. Week-by-week miscarriage rates see a sharp reduction after 12 weeks of gestation^38^. Women and parents often decide to tell friends following week 12^39^, which may result in recommendations to use apps by friends. The third peak may relate to the beginning of the third trimester, specific pregnancy examinations, increased preparation for birth and the puerperium, or the passing of the periviable period^40^. No push notifications were sent by the application to promote the use of the pregnancy tracker or the application.

### Most users use the symptom tracker more than once, but only on one day

The characteristics of the data sets in Table 1 show that most users report more than one symptom, as the characteristics of the data sets “all users” (selection criterion A, 183,732 users, 1,549,186 symptoms) and “tracked at least two symptoms” (selection criterion D, 168,225 users, 1,533,679 symptoms) are similar. User and symptom counts decrease dramatically when only data from those users who used the tracker on at least two different days are considered (selection criterion G, 38,396 users, 785,717 symptoms). Consequently, only 20.9% of users open the symptom tracker on two different days. If the first day of use is excluded for all users (selection criterion H), the size of the data set decreases to 38.0% of the original data set size (588,533 of 1,549,186 symptoms). If symptoms from the first tracker use are excluded (selection criterion F), 99,838 users with 942,481 tracked symptoms remain. Thus, only 54.3% of users are using the symptom tracker at least twice.

Possible explanations are that most users try the symptom tracker but are not interested in using it for a longer period of time or that the tracker does not appeal to them.

### Single tracker use could lead to invalidate data

When the tracker is used immediately after registration, it is also unclear whether users are actually suffering from a symptom or arbitrarily record symptoms to simply test the system. This raises questions about the validity of the overall data. This was the reason for evaluating different data set selection criteria. It is also important to note that this phenomenon contributes to data sparsity, which must be taken into account in the subsequent evaluation.

### The differences between single-day and very active users are negligible

To investigate the aforementioned validity, we plotted the weekly symptom occurrence for different data selection criteria in Fig. 2. Data from the first tracker use (selection criterion B) consistently show a lower occurrence than all other data selection criteria. The occurrences derived from the remaining data selection criteria have more similar characteristics. The overall trend in the data from the first tracker use by the user (selection criterion B) is in line with the data from other data selection criteria.

Statistical differences were evaluated between users that tracked on one day only (selection criterion E) and users that tracked on two or more days (selection criterion G). These groups are mutually exclusive. 45% of the symptom-week-pairs showed a statistical difference. This can be explained by the large sample sizes. Literature interprets the observed effect sizes (*ϕ*: 0.03 ± 0.02, odds ratio: 1.07 ± 0.2, risk ratio: 1.05 ± 0.16, mean ± standard deviation) as small^41,42^. Thus, although statistical differences exist, we see the differences between the two groups as negligible.

### Users initially test the system with a few correct symptoms, but report additional symptoms in direct succession

The reported occurrence of symptoms is too low when only data from the first tracker use are considered (selection criterion B). A low number of reported symptoms on the first tracker use could explain this. However, a user’s first symptom report is the one with the highest mean count of reported symptoms. Instead, we suspect that many users make another symptom report in direct succession to their first. This is based on the observation of 606,705 reported symptoms on first tracker use (selection criterion B), but 960,653 reported symptoms on the first day of usage (selection criterion C). Similarly, the usage time, i.e., the difference between the first and last use of the symptom tracker in the respective data set, is short (0.00 ± 0.03 days, mean ± standard deviation, Table 1, selection criterion C).

### Sparse data makes occurrence estimation difficult and unreliable for large time periods

Table 2 shows large differences between the symptom occurrences of the different data selection criteria. Occurrences increase with the strictness of the selection criteria. They are high for the data set with the most active users and low when all users are included.

This is due to sparse data and successive underreporting of symptoms among users with a small number of reports. For example, suppose a user reports symptoms only at week 8 of pregnancy. If they did not experience back pain in week 8, but instead in week 26, this symptom is not captured. The mean occurrence across the whole pregnancy duration is too low.

In summary, with increasing sparsity of data and longer evaluated time periods, the occurrence of symptoms becomes increasingly underreported. This implies an analysis at smaller time intervals.

### Weekly symptom occurrence estimation is an adequate method for symptom progress analysis, and the only means available for analysis of sparse data

Note that the expressiveness differs, i.e., a weekly occurrence of water retention in pregnancy week 38 of ~37% is only a lower bound for overall occurrence across pregnancy: Those patients that did not experience this symptom in week 38 may have experienced it in week 39, so the overall occurrence can be higher.

The appropriateness of the approach we chose (time-period-based occurrence estimation at the gestation week level) is emphasized by the stability and similarity of the symptom occurrence between gestation weeks. If the approach were not adequate, the curves in Fig. 2 would show more noise and differences between successive gestation weeks. Instead, overall trends are clearly visible. Larger differences between consecutive weeks occur in the first 4 weeks of pregnancy, especially after applying data selection criteria with a small number of users (e.g., selection criterion N). This is due to the low number of reporting users in the first weeks of pregnancy and lower explanatory power.

### Time frame and time of reporting lead to incomparability of symptom prevalences

Table 2 shows symptom prevalences for different data selection criteria. As discussed before, sparse data are inappropriate for prevalence estimation over the entire gestational period. Data from users active over at least 5 months (selection criterion N) is denser and the best available choice for comparison with the literature.

Previous works found vast differences in symptom prevalences across different literature sources^43–47^. Several reasons have been suggested as causes: The use of different study settings (e.g., retrospective, prospective studies), symptom criteria (e.g., self-reported, based on clinical history, based on clinical examination), insufficient differentiation between diseases (e.g., between lower back pain and pelvic girdle pain), and choice of measurement tool (e.g., validity of questionnaires)^45,48^.

Absolute symptom prevalences are not comparable across different time frames or time points: In our work, the highest reported weekly occurrence of back pain is 54% at 28 weeks’ gestation. At the same time, the occurrence throughout pregnancy is 93%. One study asked for symptoms in the past 3 months at three different times in pregnancy, resulting in a prevalence of 69% at 28 weeks and 68% at 36 weeks^7^. In another prospective study, women were interviewed at the end of each trimester of pregnancy and 6 weeks postpartum whether they could recall any symptoms in the preceding trimester. This resulted in an overall prevalence of 48%, with 63% in the second trimester, 76% in the third, and 39% in the puerperium^3^. Other work asked about the frequency of symptoms “in the last month”, resulting in a total prevalence of 60%. However, the questionnaire was not issued at a set point in time; rather, some participants were in their second, others in their third trimester^4^. This is aside from any additional effects that may be caused by retrospective analysis of questionnaires.

Furthermore, symptom prevalence is not necessarily of interest, but may be rather misleading. The high occurrence of back pain in our data only indicates that almost all women suffer from back pain, but does not provide information about severity or frequency. Larger time spans increase the likelihood that prevalences are elevated by chance. Instead, symptom frequency, time course, and severity may be of greater interest.

### More research is needed to establish symptom occurrence as a surrogate parameter for symptom prevalence

As presented before, the similarity of neighboring weekly occurrence in Fig. 2 can be used as an argument for data validity. However, as outlined in the previous paragraph, our occurrences cannot be compared with findings from literature due to different time frames and reporting times. However, such a comparison is necessary to evaluate the validity of the symptom occurrence as a surrogate parameter for symptom prevalence. In order to perform such a validation, close attention should be paid to the data density in the respective time frames. For example, if data are compared on a week-by-week basis (as in Fig. 2), sparse data sets can be used, as long as the subsets used for weekly analysis are sufficiently dense. If validity is evaluated at larger time frames (e.g., throughout the whole pregnancy, as in Table 2), data must be sufficiently dense throughout the whole duration of pregnancy.

### Symptom course in literature

Our results in Fig. 2 suggest that the occurrence of back pain is more continuous, with week-by-week occurrence peaking at about 24–26 weeks’ gestation. Pelvic pain peaks at gestational week 38. A review by Casgrande et al. reports that low back pain begins in the second trimester, averaging around 22 weeks’ gestation, and affects between 20% and 90% of women, whereas most studies report a prevalence above 50%^44^. The work by Vermani et al. described that low back and pelvic girdle pain begins around 18 weeks of gestation and peaks between 24 and 36 weeks of gestation^43^. Both papers described that pelvic girdle pain peaks between 24 and 36 weeks of gestation.

Incontinence was found less prevalent in early than in late pregnancy, which is in line with our results^47^.

Our data suggests a rapid increase of breathlessness in the first trimester to about 30% weekly occurrence. The highest weekly occurrence was found around 32 weeks of gestation, with 40% weekly occurrence. Other works have distinguished between breathlessness at rest and during exercise^3,5^. One review found activity-related breathlessness to be prevalent in 75% of pregnant women by ~30 weeks gestation, and stated that severity widely varies^49^. Severity or quality of breathlessness were not captured by the symptom tracker.

We could not find comprehensive reviews of the prevalence and progress of constipation or diarrhea. Our data show that occurrence is highest around 9–12 weeks of gestation and declines steadily thereafter. A recent work describes constipation to be higher in the first and second trimester, than in the third^50–52^. Sex hormones have been suggested as a major influence^53^.

The longitudinal changes of diarrhea presented in our data shows an increase in occurrence around gestation week 6 and toward the end of pregnancy. Diarrhea has been described as a precursor to labor, but also as a potential symptom of more serious causes^51^. Earlier work suggested that there is no convincing evidence that the pathogenesis of diarrhea in pregnant women differs from that of the non-pregnant population and that pregnancy promotes constipation rather than diarrhea^54^. More recent work suggests pregnancy-related factors, such as hormonal changes, dietary changes, or preexisting conditions^55^. Our work supports the latter.

Our results clearly show three phases of fatigue: An early peak of high weekly occurrence around gestation weeks 7 and 8, a trough around gestation week 21, and slightly elevated levels toward the end of the pregnancy. No clear picture of the progress of fatigue across pregnancy exists: Previous work found no significant differences in fatigue severity between pregnancy trimesters^56^. Other work found statistically significant higher fatigue levels in the first and third trimesters, compared to the second trimester^57^. This also applies to prevalence^3,58^. Other work found an increase from early to late pregnancy^59,60^, while others found the opposite^61^. Prior works may not have used sufficiently fine-grained time periods for analysis and thus were unable to capture these longitudinal changes.

We found a steadily increasing weekly occurrence of foot pain, peaking just before delivery. This is consistent with reports from the literature and can be explained by weight gain, shift of center of mass, and changes in biomechanics^62,63^.

The course of headache shows a distinct spike in headache occurrence at gestation week 16, and a small increase in headache occurrence shortly before birth. Migraine and tension-type headaches are the most common primary headache types during pregnancy. A systematic review reports that most women who suffer from migraine experience marked improvement during pregnancy, with a significant reduction in frequency and intensity, if not complete resolution^64–67^. One study only found a reduction in the third semester^68^. Another study described an increase in headache burden four weeks before delivery in multiparous women^69^. The onset of migraine during pregnancy is relatively uncommon and poorly understood, with incidences between 1.8% and 18%^65^. Overall, we did not find data on the longitudinal progress of weekly headache prevalence across pregnancy previously reported in literature. Our data set does not distinguish between different headache types and does not include information on migraine history. The small increase in headache occurrence shortly before birth may support prior work^69^.

For heartburn, our data indicate a continuous increase in weekly occurrence, peaking between gestational weeks 32–36. Some studies have found that heartburn increases throughout pregnancy, whereas other studies suggest the opposite^70^.

Nausea peaks with around 60% weekly occurrence at approximately gestation week 8 in our data. Previous work has found that nausea is prevalent in more than 80% of women, peaks around gestational weeks 4–12, and thereafter 2.5–10% of women experience it^71–74^. This is clearly in line with our results.

Our work clearly outlines increased sleeping difficulties in the third trimester. A previous scoping review on sleep health in pregnancy found that sleep in each trimester is highly variable between women, but did not support the notion of large changes in sleep during pregnancy in healthy women^75^. In addition, it was found that there is a lack of data on perceived sleep satisfaction/quality.

### Symptom Correlation

We analyzed correlations between symptoms in the different trimesters as well as the Puerperium. The largest correlation (PCC: 0.54) was found between Fatigue and Nausea in the first trimester. This correlation has been reported by several prior works^76,77^.

Several strong correlations exist in the Puerperium. The involved symptoms (back pain, neck pain, incontinence, breathlessness, sleeping difficulty) may be linked to the physiological changes after birth. The correlation between headache and neck pain also exist in other trimesters (PCC: 0.32–0.43). Fatigue and sleeping difficulties are moderately associated in all trimesters (PCC: 0.38–0.41). Furthermore, headache and nausea were correlated in the first trimester (PCC: 0.4).

We can confirm previous reports on an association between back pain and girdle pain using the PCC in the first trimester (Theirs: 0.33, ours: 0.25)^1^.

### Symptom Tracker Validity

**Fig. 3:**
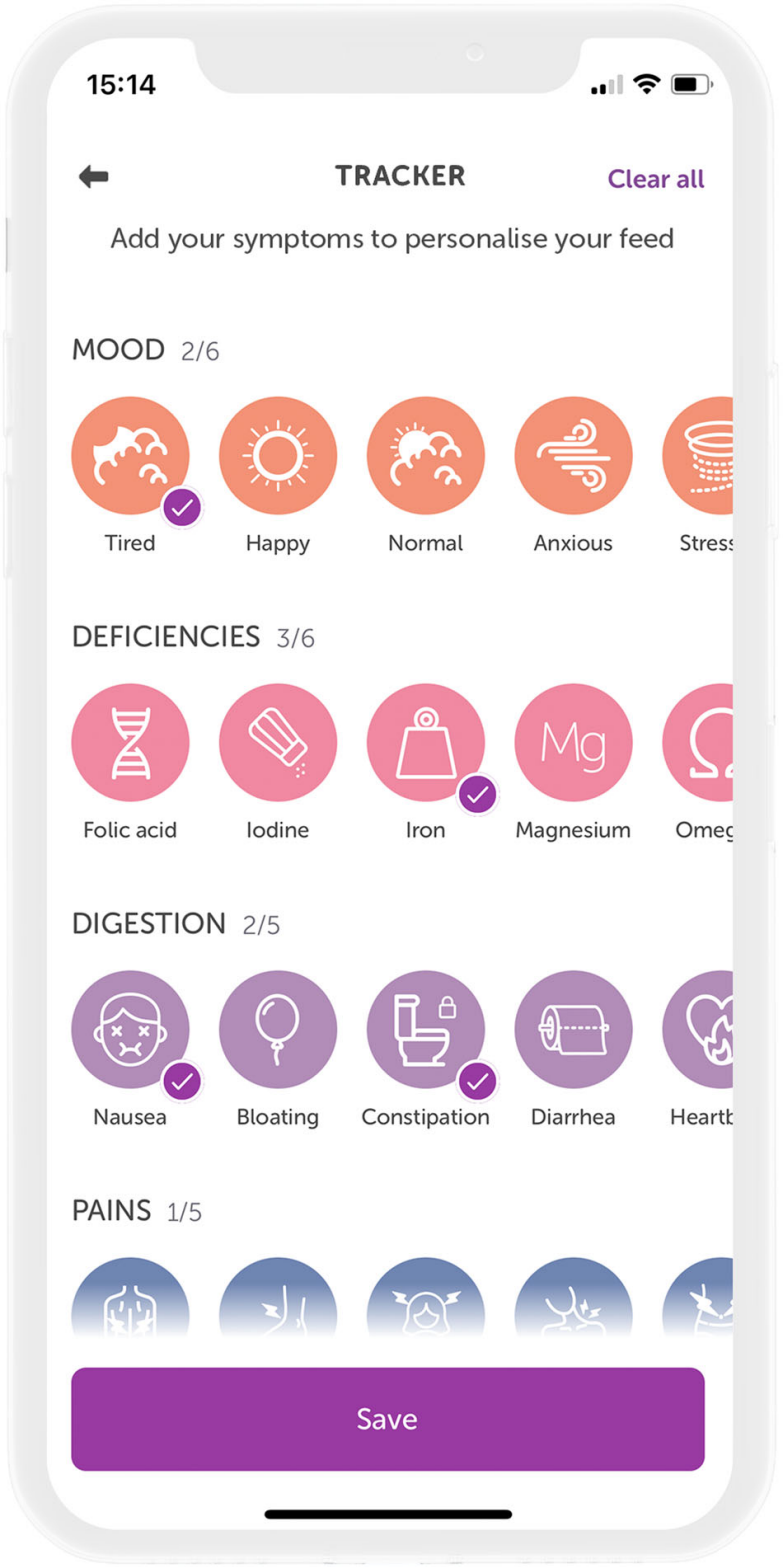
Screenshot of the symptom tracker in the Keleya pregnancy app. Some symptoms require a “left swipe” to be accessible.

This study reports real-world data, generated through a symptom tracker used as patient-reported outcome measure. We found no previous works that investigated the accuracy and validity of symptom trackers such as the one used to collect the data analyzed in this work. Previous work has mostly focused on multi-page symptom checkers or questionnaires^78–80^. Pregnancy apps often use more simplified, iconified, single-page symptom trackers. Figure 3 shows an image of the symptom tracker used for this work.

For symptoms for which there is already extensive work (e.g., nausea), our results are similar to findings in the literature, which gives hope that these trackers can provide some level of validity. Due to a lack of ground truth, more detailed conclusions cannot be made.

Some users started tracking symptoms early. Figure 1 shows ~1000–3000 symptoms reported weekly at gestation weeks 1–3. These data may be incorrect. One possible explanation is incorrect data entry (e.g., entering incorrect ovulation data used to calculate the week of pregnancy). However, the data may also be of natural and correct origin. For example, these data may originate from women using reproductive technologies or those tracking pregnancy signs using ovulation predictor kits or home pregnancy tests^81^. In 2020, 12,503 children were born in Germany (total births: 773,144, 1.6%) using assisted reproductive technologies^82,83^. We, therefore, decided to include these data.

Visible spikes in Fig. 2, especially in the “users that tracked symptoms in at least 5 different months” group (selection criterion N), can be attributed to a small number of users. Details on the time-period-based occurrence estimation are provided in the Methods section.

### Limitations

Our work and its data set are subject to several limitations. Most arise from the data set, which was not primarily intended to generate new scientific insights. The main limitation is that the data set does not contain information about clinical pregnancy outcomes. This information is not queried from users. The symptom tracker only provides data on occurrence, but not on the severity or quality of the reported symptoms. In addition, symptoms can have different dimensions, such as fatigue, which can refer to different body parts^84^. Furthermore, the symptom tracker does not cover all symptoms suggested by previous work^4^.

To take full advantage of the data set size, most of our analysis was conducted cross-sectionally for all users (selection criterion A). A first longitudinal analysis was performed in the form of a correlation analysis on the data of those users who were active for at least 5 months (selection criterion N). An initial application of unsupervised machine learning techniques for clustering did not yield meaningful results. We will use more advanced methods in the future.

The order of symptoms on the symptom tracker screen is not randomized, which may cause unwanted side effects. Furthermore, some symptoms are only accessed after a “right swipe” on the symptom tracker screen. It is unclear whether this affects the reporting frequency of those symptoms. No information is available on the impact of usability on adequate symptom tracking, as well as symptom tracker validity.

Foot pain and flatulence were added to the symptom tracker during the investigated period and were not available prior to these dates. While the overall symptom progress can still be analyzed, the occurrence estimation is incorrect, and we have marked these results as unreliable. Similarly, the application underwent continuous development during the investigated period. This may have had an impact on the usage of the pregnancy tracker.

Lastly, the data collected in this work were generated by a German pregnancy app, and thus the data were generated mainly by women in Germany. Keleya operates on a freemium model. The app is reimbursed by some health insurances in German. Nonetheless, the need to pay for some features may impact the user base. The demographics of app users may therefore not be generalizable to society as a whole. This may promote health inequity if derived research findings are used without considering this aspect.

### Implications

In summary, this work is subject to limitations, which originate in the secondary use of tracker data from a commercial pregnancy app. On the other hand, these real-life data offer an unprecedented data quantity, that exceeds previous work by orders of magnitude. It is the first study to extensively evaluate symptom progress in a longitudinal fashion. It is not subject to several reporting shortcomings of prior work: We are able to include early pregnancy weeks and no retrospective data collection bias is present^29^.

This allows our work to contribute in several ways: We presented new insights on symptom progress in several cases, inadequate reporting of time span and time of reporting as additional explanations for prevalence differences in literature, and lastly an overall approach for analysis of sparse symptom reporting data.

Close cooperation between academia and industry is desirable to maximize benefits for all parties. For best cooperation, it is important to understand the needs, requirements, and goals of all stakeholders. These include pregnant women (in some cases patients) as users, their relatives/friends, application manufacturers and their developers, as well as researchers.

Consistent with user-centered design, user aims, requirements, motivation, and benefit should always come first. However, the use of pregnancy apps has implications for users. It may carry risks and entails privacy considerations, such as the use and potential misuse of data and the corresponding privacy implications. Data minimization as principle may be able to reduce these risks. On the other hand, users and pregnant women in general can benefit from better care and new scientific insights. Mobile health has the potential to improve care. Mobile health has a particularly high potential to improve care in developing countries^85^. Users are able to learn from their own data, feel and are more empowered, increase their health literacy^86^. It is important to keep equity and diversity in mind, as outlined in the limitations of this work, in order to make these benefits available to everyone. The use of frameworks and guiding principles can help to increase health equity^87,88^.

Researchers need to understand that application developers are subject to commercial constraints. Likewise, they need to understand the development process of applications. Although the primary aim for both parties is to maximize the benefit for the user, this goal may be interpreted differently by each side. Developers may aim to maximize user needs, requirements, and demands. Scientific validity of the data generated may only be a secondary goal. Unlike in most medical studies, measurement devices, and thus the data collected, can change as a result of continuous development. In addition, researchers must accept that measurement instruments (such as the symptom tracker used in this work) may offer lower validity or detail compared to traditional instruments such as comprehensive, validated questionnaires. Users may not be motivated to complete extensive questionnaires.

Application developers can integrate basic features to support research. Sociodemographic data, even if only in a basic form, offer new insights into disease trajectories. Information on basic pregnancy outcomes would be helpful, such as date of birth, birth weight, and mode of delivery. A separate section for citizen science or data donation could be an adequate form for requesting these data.

Developers should ensure that they at least capture any symptoms known to be associated with pregnancy outcomes. This includes nausea, insomnia, vaginal bleeding, vomiting, and headache. On the other hand, they should avoid incorporating features for which no clear evidence exists, such as kick or fetal movement counting^89^. Overall, industry benefits from a large user base, and academia subsequently from good data availability.

This combination of factors (user demographics, reported symptoms, outcomes) may enable new research in the future and create new large data sets for prospective analysis by machine learning.

Some user-tailored recommendations based on symptom tracker use can already be implemented: Reported vaginal bleeding between weeks 6 and 8 could prompt a suggested physician visit^20^. Reporting headache without prior history could lead to additional prompts, for example, based on “red flags” for headache in pregnancy^64,90^. Severe outcomes aside, symptom-based responses can also be used to inform pregnant women about treatment options for conditions for which women are reluctant to seek help, such as stress incontinence^47,91^.

It is important to note that functionalities must be integrated with care and close assessment and evaluation (as in initial clinical trials) is necessary to avoid adverse effects, unnecessary panic, or anxiety^92^.

Overall, real-life symptom tracker data provide a large data quantity, while enabling longitudinal analysis. Future work should aim to investigate the quality, severity, genesis, and phenotypes of symptoms and their relationship to outcomes.

## Methods

### Keleya pregnancy app

The presented data originates from the symptom tracker of Keleya, a pregnancy app. The app is available in Google Play (Android) and Apple App Store (iOS). A screenshot of the symptom tracker is shown in Fig. 3. The tracker is divided into several categories. The deficiency category is not analyzed in this work. Only fatigue (tired) was analyzed in the mood category. Nausea, flatulence (bloating), constipation, diarrhea, and heartburn were shown as part of the digestion group. The pain group included back pain, foot pain, headache, neck tension, and pelvic area pain. Breathlessness, incontinence (bladder weakness), sleeping difficulty, and water retention were included in the complaints group. The app manufacturer did not include symptoms that are highly likely to indicate adverse events to avoid users simply reporting the symptom in the app but not consulting their caring medical professional. No free text symptom input is possible. Tracking symptoms has an impact on the workouts and recipes suggested by the application, for example if nausea or heartburn are tracked. Users reporting headaches will not be shown exercises involving a “head down” position.

### Data extraction and processing

A written data sharing agreement between the parties was in place for data use. Each reported symptom is stored in a relational database, with one entry per symptom, including a time stamp. If several symptoms are reported at the same time (in “one report”), each symptom is stored in its own row, featuring the same time stamp. Consequently, if the symptoms are evaluated in the smallest possible time span, symptoms are considered co-occuring if their time stamps match.

The production data is stored in a relational database. Data were anonymized and exported as *.csv-file for consecutive processing and analysis. All processing and analysis steps were performed using Python 3.9.1, Pandas 1.2.0 was used for data management, Matplotlib 3.6.2 to generate graphs, and Scipy 1.10.1 as well as Statsmodels 0.13.5 for statistical analysis.

As preprocessing step, data recorded after pregnancy week 50 were excluded. Data after gestation week 41 are not included in Fig. 2, as weekly symptom counts were too low (<10 in some cases) to be expressive. Trimester 1 was defined as weeks 0–13, trimester 2 as weeks 14–27, and trimester 3 from weeks 28–40. As the exact birth date of pregnancies is not known, data was assigned to the Puerperium if symptoms were reported from weeks 41–50.

We excluded all “deficiency” categories, as they are not present in similar works^1–4,6–9^, and it is unclear how pregnant women decide that these symptoms affect them. These symptoms are denoted and grouped as “Nutrition Deficiencies” in selected tables and analysis.

The data selection criteria presented in Table 1 were collectively brainstormed and discussed by the authors. As little previous work in literature exists on this topic, ideas from commercial web usage analytics tools were adapted, such as tracking frequency or a minimum usage time. We furthermore differentiated the data selection criteria by new and returning users.

The registration date is the date at which the user registered an account for the underlying Keleya pregnancy app. The usage time is defined as the difference between the first and last day of usage of the symptom tracker. Note that the registration time can differ from the first day of usage.

Two symptoms were added during the investigated time frame: Foot pain (March 6, 2020) and flatulence (June 20, 2020). The resulting occurrence calculations are thus unreliable.

### Analysis and statistical evaluation

Symptom occurrence was calculated as the number of users affected by a symptom divided by all users that reported a symptom in the respective time period. In fact, if five users reported back pain in gestation week 10, and 20 users reported symptoms in total in this period, the occurrence is 25%.

The week-by-week symptom occurrence can be seen as a Bernoulli experiment. Users experience or do not experience a symptom. We thus used the Agresti-Coull interval to estimate the 95% confidence intervals, using the *proportion_confint* function of the Statsmodels package. The Agresti-Coull interval was suggested by prior work for large samples sizes^93^.

Chi-squared tests were performed using the *chi2_contingency* function of the Scipy package. A *p* value of 0.05 was used as threshold for significance analysis. For calculating effect sizes, we defined $\phi =\sqrt{({\chi }^{2})/N}$, where *χ*^2^ is the respective statistics, and *N* the total number of samples. Odds ratios were calculated using Scipy’s *odds_ratio* function, and the risk ratio was derived by dividing the two occurrences.

To assess correlation coefficients, the data set with the most active users (selection criterion N) was used. For each user, the respective symptom counts in each time frame (Trimesters 1–3, Puerperium) were calculated. This resulted in an array of size 84 for each user (21 symptoms for 4 time frames). Data was normalized user-wise across all time frames to accommodate for differences in reporting, e.g., where one user reported 50 symptoms in total, and another 500 in total. Thus, each symptom-period count was normalized by the total sum of reported symptoms by this user. From this, the total sum of all reported symptoms for each individual user is 1 after normalization.

### Supplementary information

Supplementary Material

## Data Availability

The data sets used and/or analyzed during the current study available from the corresponding author on reasonable request and approval from Keleya Digital-Health Solutions GmbH.

## Code availability

The underlying code for this study and data sets is not publicly available but may be made available to qualified researchers on reasonable request from the corresponding author.
