# Supplementary material for: Prevalence and course of pregnancy symptoms using self-reported pregnancy app symptom tracker data
Source: NPJ Digit Med. 2023 Oct 11;6:189. doi: 10.1038/s41746-023-00935-3 (PMC10567694; doi:10.1038/s41746-023-00935-3)
Supplement: Supplementary file 1 — Supplementary Material [file 41746_2023_935_MOESM1_ESM.pdf]

Supplementary Table 1: Detailed Occurrence Data

Table 1 Symptom total counts, reporting percentages and frequency. Columns depict statistics for the full duration of pregnancy (trimesters 1-3), for each individual trimester, and the postpartum period.

Symptom		Pregnancy	First Trimester	Second Trimester	Third Trimester	Postpartum
Back Pain	T^1	20,305 (9.04%)	2,822 (6.09%)	9,273 (10.04%)	8,210 (9.57%)	223 (11.08%)
	O^2	2,702 (92.57%)	1,184 (53.55%)	2,300 (79.28%)	2,012 (72.92%)	143 (47.35%)
	F^3	7.51 ± 8.4	2.38 ± 2.8	4.03 ± 4.5	4.08 ± 5.2	1.56 ± 1.2
Bladder Weakness	T	7,150 (3.18%)	470 (1.01%)	2,208 (2.39%)	4,472 (5.21%)	100 (4.97%)
	O	1,551 (53.13%)	234 (10.58%)	818 (28.20%)	1,260 (45.67%)	72 (23.84%)
	F	4.61 ± 6.5	2.01 ± 2.1	2.7 ± 3.8	3.55 ± 5.0	1.39 ± 1.0
Breathlessnes	T	12,905 (5.75%)	2,453 (5.29%)	5,335 (5.77%)	5,117 (5.96%)	47 (2.34%)
	O	2,365 (81.02%)	1,025 (46.36%)	1,781 (61.39%)	1,599 (57.96%)	34 (11.26%)
	F	5.46 ± 6.7	2.39 ± 2.7	3.0 ± 3.0	3.2 ± 4.9	1.38 ± 0.85
Constipation	T	10,782 (4.80%)	2,711 (5.85%)	4,863 (5.26%)	3,208 (3.74%)	103 (5.12%)
	O	2,223 (76.16%)	1,174 (53.10%)	1,617 (55.74%)	1,116 (40.45%)	66 (21.85%)
	F	4.85 ± 6.1	2.31 ± 2.1	3.01 ± 3.1	2.87 ± 4.9	1.56 ± 1.3
Diarrhea	T	3,976 (1.77%)	1,091 (2.35%)	1,388 (1.50%)	1,497 (1.74%)	45 (2.24%)
	O	1,430 (48.99%)	595 (26.91%)	727 (25.06%)	715 (25.92%)	30 (9.93%)
	F	2.78 ± 3.5	1.83 ± 1.5	1.91 ± 1.8	2.09 ± 2.9	1.5 ± 0.97
Fatigue	T	19,623 (8.74%)	5,484 (11.83%)	7,301 (7.90%)	6,838 (7.97%)	179 (8.90%)
	O	2,711 (92.87%)	1,711 (77.39%)	2,019 (69.60%)	1,812 (65.68%)	130 (43.05%)
	F	7.24 ± 9.1	3.21 ± 3.8	3.62 ± 4.6	3.77 ± 6.1	1.38 ± 0.84
Flatulence[4]	T	7,466 (3.32%)	2,278 (4.91%)	3,053 (3.30%)	2,135 (2.49%)	35 (1.74%)
	O	1,452 (49.74%)	821 (37.13%)	996 (34.33%)	684 (24.79%)	23 (7.62%)
	F	5.14 ± 7.9	2.77 ± 3.1	3.07 ± 4.0	3.12 ± 6.6	1.52 ± 1.1
Foot Pain[4]	T	2,920 (1.30%)	209 (0.45%)	930 (1.01%)	1,781 (2.08%)	27 (1.34%)
	O	771 (26.41%)	129 (5.83%)	387 (13.34%)	550 (19.93%)	21 (6.95%)
	F	3.79 ± 5.7	1.62 ± 1.3	2.4 ± 3.0	3.24 ± 5.4	1.29 ± 0.64

[1] Total count of symptom reports (relative to total reported symptoms in this time frame).

[2] Occurrence (percentage) of symptom (relative to all unique users that reported a symptom in this time frame).

[3] Frequency of symptom for users that reported symptom at least once in this time frame, mean ± standard deviation.

[4] These symptoms were added during the investigated period. Occurrences are unreliable and only indicate a lower bound.

Table 1 continued

Headache	*T*	9,043 (4.03%)	2,393 (5.16%)	4,498 (4.87%)	2,152 (2.51%)	91 (4.52%)
	O	2,123 (72.73%)	1,058 (47.85%)	1,635 (56.36%)	832 (30.16%)	78 (25.83%)
	F	4.26 ± 5.5	2.26 ± 2.6	2.75 ± 3.0	2.59 ± 4.2	1.17 ± 0.54
Heartburn	*T*	9,834 (4.38%)	1,225 (2.64%)	3,647 (3.95%)	4,962 (5.78%)	66 (3.28%)
	O	2,014 (69.00%)	597 (27.00%)	1,293 (44.57%)	1,489 (53.97%)	41 (13.58%)
	F	4.88 ± 6.9	2.05 ± 2.0	2.82 ± 3.2	3.33 ± 5.7	1.61 ± 1.8
Incontinence	*T*	5,462 (2.43%)	504 (1.09%)	2,287 (2.48%)	2,671 (3.11%)	66 (3.28%)
	O	1,332 (45.63%)	302 (13.66%)	863 (29.75%)	843 (30.55%)	50 (16.56%)
	F	4.1 ± 6.9	1.67 ± 1.7	2.65 ± 3.1	3.17 ± 6.0	1.32 ± 0.79
Mood: Happy	*T*	12,232 (5.45%)	2,224 (4.80%)	5,978 (6.47%)	4,030 (4.70%)	116 (5.77%)
	O	2,161 (74.03%)	948 (42.88%)	1,701 (58.63%)	1,264 (45.81%)	83 (27.48%)
	F	5.66 ± 8.9	2.35 ± 3.1	3.51 ± 5.3	3.19 ± 4.6	1.4 ± 0.95
Mood: Normal	*T*	16,767 (7.47%)	3,234 (6.98%)	7,753 (8.39%)	5,780 (6.74%)	127 (6.31%)
	O	2,598 (89.00%)	1,335 (60.38%)	2,091 (72.08%)	1,642 (59.51%)	91 (30.13%)
	F	6.45 ± 9.0	2.42 ± 3.2	3.71 ± 4.8	3.52 ± 5.2	1.4 ± 0.89
Mood: Scared	*T*	4,108 (1.83%)	1,124 (2.42%)	1,354 (1.47%)	1,630 (1.90%)	43 (2.14%)
	O	1,345 (46.08%)	582 (26.32%)	689 (23.75%)	721 (26.13%)	33 (10.93%)
	F	3.05 ± 4.8	1.93 ± 2.0	1.97 ± 1.6	2.26 ± 5.2	1.3 ± 0.95
Mood: Stressed	*T*	5,205 (2.32%)	1,097 (2.37%)	2,447 (2.65%)	1,661 (1.94%)	52 (2.58%)
	O	1,672 (57.28%)	631 (28.54%)	1,120 (38.61%)	789 (28.60%)	41 (13.58%)
	F	3.11 ± 3.2	1.74 ± 1.5	2.18 ± 1.9	2.11 ± 2.0	1.27 ± 0.45
Mood: Swings	*T*	7,933 (3.53%)	2,036 (4.39%)	3,286 (3.56%)	2,611 (3.04%)	72 (3.58%)
	O	2,051 (70.26%)	973 (44.01%)	1,372 (47.29%)	1,105 (40.05%)	60 (19.87%)
	F	3.87 ± 4.1	2.09 ± 1.9	2.4 ± 2.2	2.36 ± 2.7	1.2 ± 0.51

Table 1 continued

Nausea	T	8,042 (3.58%)	4,610 (9.95%)	2,095 (2.27%)	1,337 (1.56%)	40 (1.99%)
	O	1,923 (65.88%)	1,446 (65.40%)	868 (29.92%)	602 (21.82%)	27 (8.94%)
	F	4.18 ± 5.4	3.19 ± 3.6	2.41 ± 3.3	2.22 ± 2.4	1.48 ± 0.89
Neck Pain	T	11,920 (5.31%)	2,416 (5.21%)	5,745 (6.22%)	3,759 (4.38%)	134 (6.66%)
	O	2,128 (72.90%)	998 (45.14%)	1,683 (58.01%)	1,148 (41.61%)	87 (28.81%)
	F	5.6 ± 7.1	2.42 ± 2.7	3.41 ± 4.1	3.27 ± 4.6	1.54 ± 1.0
Nutrition Deficiencies	T	25,873 (11.52%)	4,594 (9.91%)	10,382 (11.24%)	10,897 (12.70%)	243 (12.08%)
	O	2,394 (82.01%)	961 (43.46%)	1,773 (61.12%)	1,755 (63.61%)	101 (33.44%)
	F	10.8 ± 1.7e+01	4.78 ± 5.4	5.86 ± 8.1	6.21 ± 1.3e+01	2.41 ± 2.9
Pelvic Pain	T	10,177 (4.53%)	931 (2.01%)	3,734 (4.04%)	5,512 (6.42%)	96 (4.77%)
	O	2,013 (68.96%)	502 (22.70%)	1,308 (45.09%)	1,431 (51.87%)	67 (22.19%)
	F	5.06 ± 7.5	1.85 ± 1.8	2.85 ± 3.3	3.85 ± 6.6	1.43 ± 0.92
Sleeping Difficulty	T	12,852 (5.72%)	2,447 (5.28%)	4,847 (5.25%)	5,558 (6.48%)	107 (5.32%)
	O	2,318 (79.41%)	985 (44.55%)	1,614 (55.64%)	1,637 (59.33%)	80 (26.49%)
	F	5.54 ± 7.1	2.48 ± 3.2	3.0 ± 3.5	3.4 ± 4.6	1.34 ± 0.93
Symptoms		224,575	46,353	92,404	85,818	2,012
Users		2,919	2,211	2,901	2,759	302

Supplementary Table 2: Spearman Correlation Coefficients

Table 2 10 largest Spearman correlation coefficients (SCC). Each symptom in each time period (trimesters, Puerperium) was correlated, using data from the most active users (selection criterion N). No negative values are included, as they were not among the largest absolute values.

Time Frame: Symptom	Time Frame: Symptom	SCC
First: Fatigue	First: Nausea	0.59
Postpartum: Back Pain	Postpartum: Fatigue	0.56
Postpartum: Back Pain	Postpartum: Neck Pain	0.53
Postpartum: Back Pain	Postpartum: Nutrition Deficiencies	0.51
Postpartum: Fatigue	Postpartum: Nutrition Deficiencies	0.49
Postpartum: Fatigue	Postpartum: Neck Pain	0.48
Postpartum: Headache	Postpartum: Neck Pain	0.47
Postpartum: Back Pain	Postpartum: Sleeping Difficulty	0.45
First: Back Pain	First: Neck Pain	0.45
Postpartum: Constipation	Postpartum: Nutrition Deficiencies	0.44

Springer Nature 2021 LaTeX template

Supplementary Figure 1: Pearson Correlation Coefficient Matrix

Pearson Correlation Coefficients (PCC) matrix, depicted as heat map.

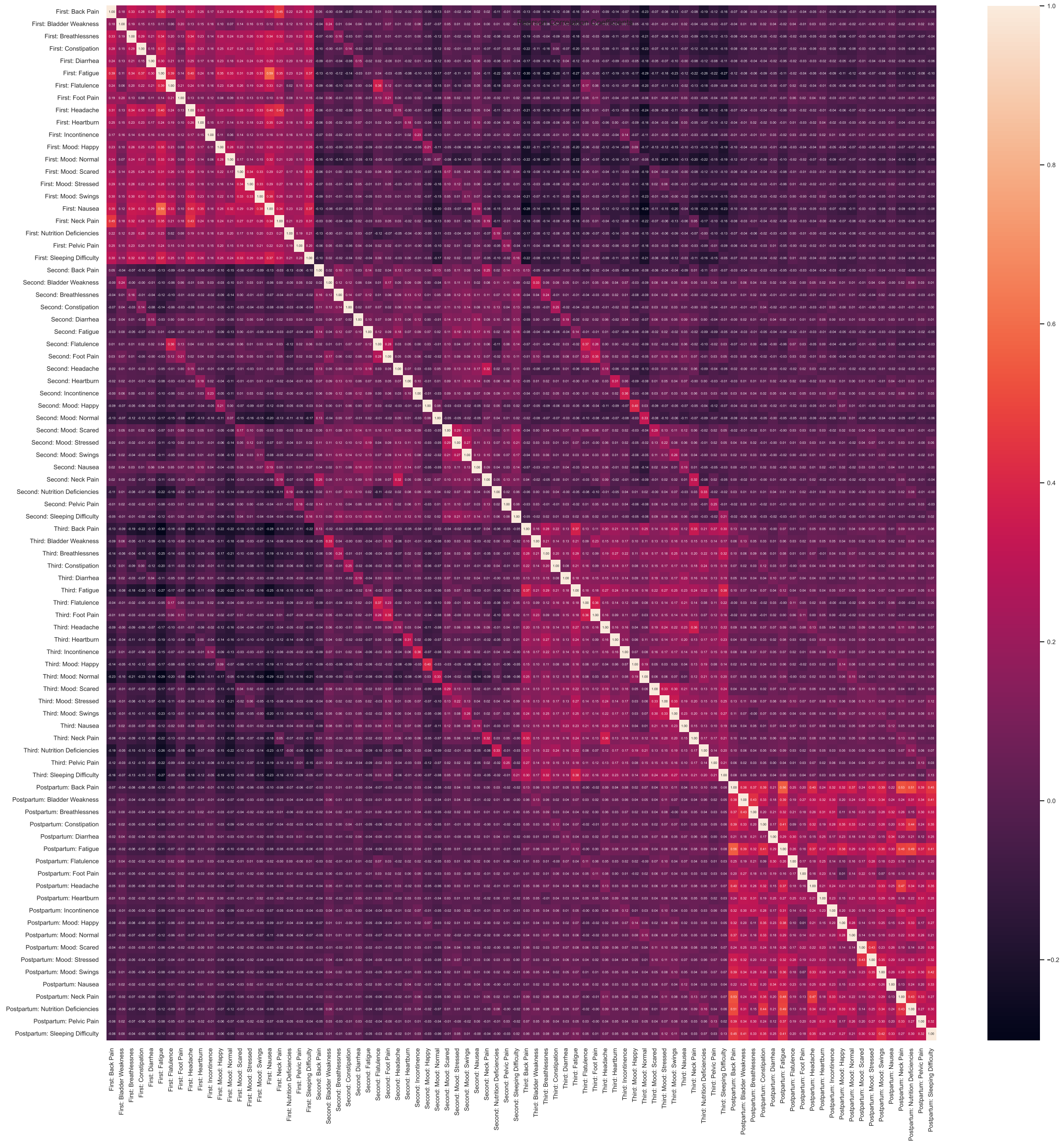
First: Back Pain
First: Bladder Weakness
First: Breathlessness
First: Constipation
First: Diarrhea
First: Fatigue
First: Flatulence
First: Foot Pain
First: Headache
First: Heartburn
First: Incontinence
First: Mood: Happy
First: Mood: Normal
First: Mood: Scared
First: Mood: Stressed
First: Mood: Swings
First: Nausea
First: Neck Pain
First: Nutrition Deficiencies
First: Pelvic Pain
First: Sleeping Difficulty
Second: Back Pain
Second: Bladder Weakness
Second: Breathlessness
Second: Constipation
Second: Diarrhea
Second: Fatigue
Second: Flatulence
Second: Foot Pain
Second: Headache
Second: Heartburn
Second: Incontinence
Second: Mood: Happy
Second: Mood: Normal
Second: Mood: Scared
Second: Mood: Stressed
Second: Mood: Swings
Second: Nausea
Second: Neck Pain
Second: Nutrition Deficiencies
Second: Pelvic Pain
Second: Sleeping Difficulty
Third: Back Pain
Third: Bladder Weakness
Third: Breathlessness
Third: Constipation
Third: Diarrhea
Third: Fatigue
Third: Flatulence
Third: Foot Pain
Third: Headache
Third: Heartburn
Third: Incontinence
Third: Mood: Happy
Third: Mood: Normal
Third: Mood: Scared
Third: Mood: Stressed
Third: Mood: Swings
Third: Nausea
Third: Neck Pain
Third: Nutrition Deficiencies
Third: Pelvic Pain
Third: Sleeping Difficulty
Postpartum: Back Pain
Postpartum: Bladder Weakness
Postpartum: Breathlessness
Postpartum: Constipation
Postpartum: Diarrhea
Postpartum: Fatigue
Postpartum: Flatulence
Postpartum: Foot Pain
Postpartum: Headache
Postpartum: Heartburn
Postpartum: Incontinence
Postpartum: Mood: Happy
Postpartum: Mood: Normal
Postpartum: Mood: Scared
Postpartum: Mood: Stressed
Postpartum: Mood: Swings
Postpartum: Nausea
Postpartum: Neck Pain
Postpartum: Nutrition Deficiencies
Postpartum: Pelvic Pain
Postpartum: Sleeping Difficulty
1.0
0.8
0.6
0.4
0.2
0.0
−0.2

Springer Nature 2021 LaTeX template

Supplementary Figure 2: Spearman Correlation Coefficient Matrix

Spearman Correlation Coefficients (SCC) matrix, depicted as heat map.

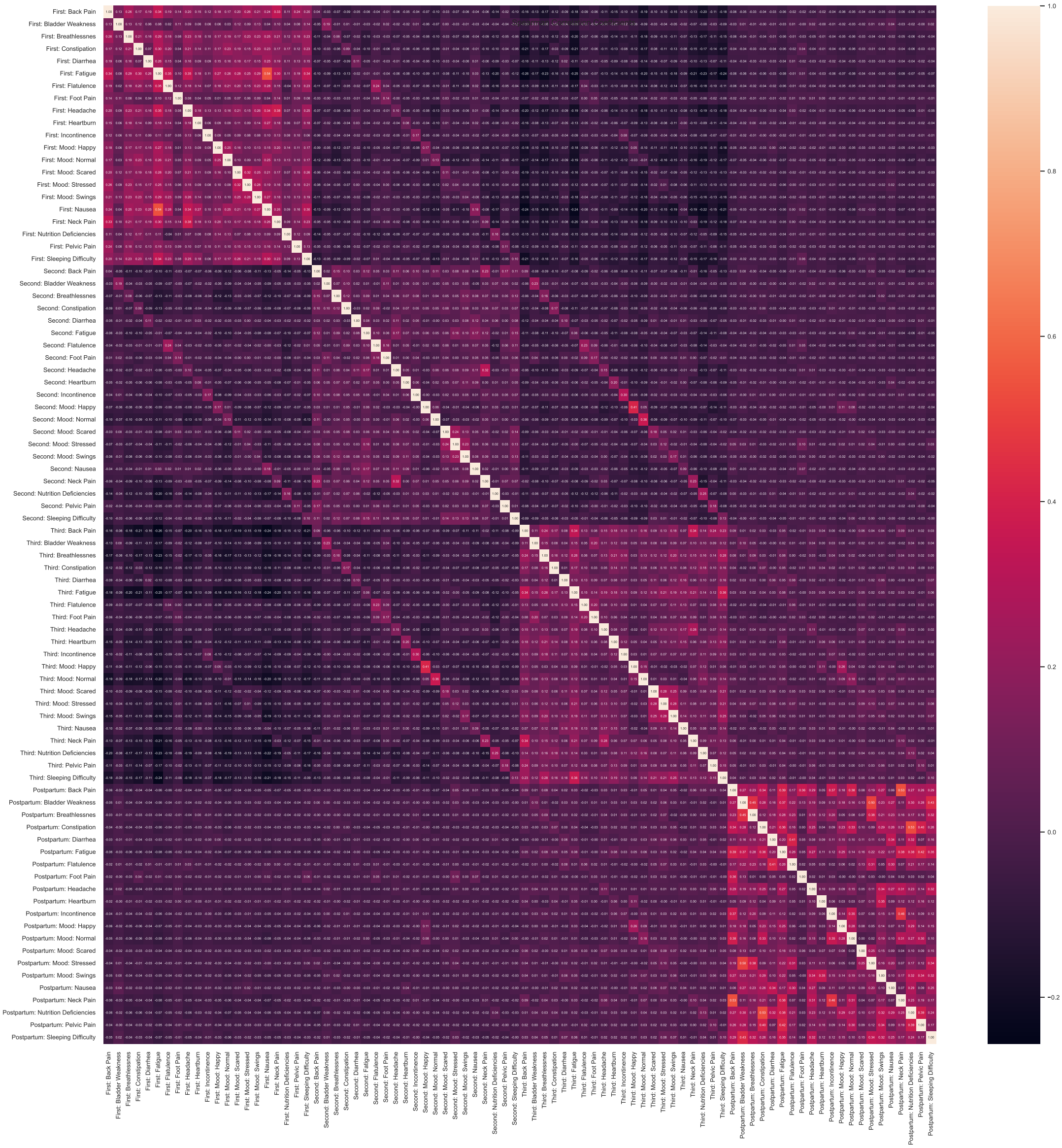
First: Back Pain
First: Bladder Weakness
First: Breathlessness
First: Constipation
First: Diarrhea
First: Fatigue
First: Flatulence
First: Foot Pain
First: Headache
First: Heartburn
First: Incontinence
First: Mood: Happy
First: Mood: Normal
First: Mood: Scared
First: Mood: Stressed
First: Mood: Swings
First: Nausea
First: Neck Pain
First: Nutrition Deficiencies
First: Pelvic Pain
First: Sleeping Difficulty
Second: Back Pain
Second: Bladder Weakness
Second: Breathlessness
Second: Constipation
Second: Diarrhea
Second: Fatigue
Second: Flatulence
Second: Foot Pain
Second: Headache
Second: Heartburn
Second: Incontinence
Second: Mood: Happy
Second: Mood: Normal
Second: Mood: Scared
Second: Mood: Stressed
Second: Mood: Swings
Second: Nausea
Second: Neck Pain
Second: Nutrition Deficiencies
Second: Pelvic Pain
Second: Sleeping Difficulty
Third: Back Pain
Third: Bladder Weakness
Third: Breathlessness
Third: Constipation
Third: Diarrhea
Third: Fatigue
Third: Flatulence
Third: Foot Pain
Third: Headache
Third: Heartburn
Third: Incontinence
Third: Mood: Happy
Third: Mood: Normal
Third: Mood: Scared
Third: Mood: Stressed
Third: Mood: Swings
Third: Nausea
Third: Neck Pain
Third: Nutrition Deficiencies
Third: Pelvic Pain
Third: Sleeping Difficulty
Postpartum: Back Pain
Postpartum: Bladder Weakness
Postpartum: Breathlessness
Postpartum: Constipation
Postpartum: Diarrhea
Postpartum: Fatigue
Postpartum: Flatulence
Postpartum: Foot Pain
Postpartum: Headache
Postpartum: Heartburn
Postpartum: Incontinence
Postpartum: Mood: Happy
Postpartum: Mood: Normal
Postpartum: Mood: Scared
Postpartum: Mood: Stressed
Postpartum: Mood: Swings
Postpartum: Nausea
Postpartum: Neck Pain
Postpartum: Nutrition Deficiencies
Postpartum: Pelvic Pain
Postpartum: Sleeping Difficulty
1.0
0.8
0.6
0.4
0.2
0.0
−0.2
